# Evaluation the level of vitamin D and its relationship with clinical symptoms in patients with COVID-19 referred to the medical center in Bam city

**DOI:** 10.3205/dgkh000512

**Published:** 2024-11-05

**Authors:** Seyed Mojtaba Mortazavi, Saeed Khoshnood, Reza Faraji, Rezvan Bagheri Baravati, Hakime Khalili, Ali Radfar, Elham Jalali, Maria Nezam Nia, Sousan Akrami, Maryam Shirani

**Affiliations:** 1 Non-Communicable Diseases Research Center, Bam University of Medical Sciences, Bam, Iran; 2 Clinical Microbiology Research Center, Ilam University of Medical Sciences, Ilam, Iran; 3 Tuberculosis and Lung Diseases Research Center, Ilam University of Medical Sciences, Ilam, Iran; 4 Pastor Educational Hospital, Bam University of Medical Sciences, Bam, Iran; 5 Student Research Committee, School of Medicine, Bam University of Medical Sciences, Bam, Iran; 6 Department of Gynecology, School of Medicine, Pasteur Hospital,Bam University of Medical Sciences Bam, Bam, Iran; 7 Department of Microbiology, Faculty of Medicine, Tehran University of Medical Sciences, Tehran, Iran; 8 Toxicology Research Center, Medical Basic Sciences Research Institute, Ahvaz Jundishapur University of Medical Sciences, Ahvaz, Iran

**Keywords:** vitamin D, COVID-19, outcome, pandemic

## Abstract

**Background:**

Vitamin D is a steroid hormone that protects against viral infections by influencing innate and adaptive immune responses. The effectiveness of vitamin D3 supplementation in COVID-19 is unknown. The study’s goal was to elucidate the relationship between blood vitamin D levels and COVID-19 clinical outcomes by examining the effect of a single high dose of vitamin D3 on the length of hospital stay in COVID-19 patients.

**Methods:**

The descriptive, retrospective study was performed from March to May 2021 at a referral center for patients with COVID-19, in Bam, Iran. A checklist consisting of demographic variables was used to gather data, and laboratory assessments of serum 25(OH) D were evaluated and documented. The connection between serum vitamin D and patient clinical outcomes was investigated after patients were given a single oral dose of 200,000 IU of vitamin D3.

**Results:**

71 COVID-19 patients were treated. Radiological results did not change substantially amongst individuals with various levels of 25(OH)D. After a single dosage of vitamin D3, mean blood levels of 25-hydroxyvitamin D increased considerably and the need for intubation and SpO_2_ decreased, and as did the respiratory rate in patients requiring hospitalization due to COVID-19.

**Conclusion:**

A single administration of 200,000 IU of vitamin D3 significantly reduced the severity of COVID-19.

## Zusammenfassung

**Hintergrund:**

Vitamin D ist ein Steroidhormon, das vor Virusinfektionen schützt, indem es die angeborene und die adaptive Immunantwort beeinflusst. Die Wirksamkeit einer Vitamin-D3-Supplementierung bei COVID-19 ist unbekannt. Ziel der Studie war es, den Zusammenhang zwischen dem Vitamin-D-Blutspiegel und der Gabe einer einzelnen hohen Vitamin-D3-Dosis auf die Krankenhausaufenthaltsdauer bei COVID-19-Patienten zu untersuchen.

**Methode:**

Die deskriptive, retrospektive Studie wurde von März bis Mai 2021 in einem Referenzzentrum für Patienten mit COVID-19 in Bam, Iran, durchgeführt. Zur Datenerfassung wurde eine Checkliste mit demografischen Variablen verwendet und der Serumspiegel von 25-Hydroxyvitamin-D (25(OH)D) bestimmt und dokumentiert. Untersucht wurde die Assoziation zwischen Serumspiegel von Vitamin D und klinischen Ergebnissen. Die Patienten erhielten eine orale Einzeldosis von 200.000 IE Vitamin D3.

**Ergebnisse:**

Es wurden 71 COVID-19-Patienten behandelt. Die radiologischen Ergebnisse veränderten sich bei Personen mit unterschiedlichen 25(OH)D-Werten nicht wesentlich. Nach einer Einzeldosis Vitamin D3 stieg der mittlere Blutspiegel von 25-Hydroxyvitamin D erheblich an. Dadurch wurden die Notwendigkeit einer Intubation herabgesetzt und der Sauerstoffpartialdruck sowie die Atemfrequenz bei Patienten, die aufgrund von COVID-19 hospitalisiert worden waren, signifikant verbessert.

**Schlussfolgerung:**

Durch einmalige Verabreichung von 200.000 IU Vitamin D3 wurde die Erkrankungsschwere von COVID-19 deutlich verbessert.

## Introduction

Because no suitable and effective therapy for COVID-19 has been developed, it is critical to identify persons who are at high risk of infection and those with a high risk of experiencing the severe form, as this allows suitable preventative actions to be implemented for these individuals. In addition, given the high number of COVID-19 patients and the collapse of many nations' healthcare systems, identifying patients who are at high risk of requiring intensive care unit (ICU) services can aid in improved patient triage and resource allocation [1]. 

Vitamin D is a hormone that controls both adaptive and innate immune responses [2]. The receptors for 25-hydroxyvitamin D (25(OH)D), the major metabolite of vitamin D in circulation, are expressed on macrophages (and dendritic cells) and are known to regulate transcription, including some genes encoding antimicrobial peptides, and may play a role in the prevention of respiratory infections. 25-hydroxyvitamin D is known to stimulate the production of the angiotensin-converting enzyme 2 (ACE-II) in the renin-angiotensin pathway, which has been found to be downregulated by SARS-COV-2. A lack of vitamin D has been linked to an increased risk of respiratory tract infections [3]. Some studies have discovered a link between vitamin D and COVID-19 severity and mortality, which is thought to be due to vitamin D's anti-inflammatory actions inhibiting a cytokine storm, a recognized pathogenic mechanism of acute respiratory distress syndrome development [4], [5]. However, concerns about residual confounding and a lack of mechanistic hypotheses for the correlation of vitamin D deficiency with negative COVID-19 results necessitate more research. Overall, data show that high vitamin D levels are related to a lower likelihood of negative COVID-19 outcomes, indicating that vitamin D may have a positive role in preventing or ameliorating the course of COVID-19 [6].

For the treatment and prevention of COVID-19 disease, cholecalciferol (vitamin D3) or calcifediol (25(OH)D) as biochemically active vitamin D metabolites have been recommended [2]. Several studies have been conducted to see whether vitamin D administration can lower COVID-19 susceptibility or severity. It has been shown that persons who receive vitamin D supplements have fewer respiratory tract infections. However, vitamin D’s immune-modulatory action is likely to be observed at 25(OH)D levels that are greater than those necessary for its skeletal effects [7]. 

Findings imply that adequate vitamin D levels may prevent COVID-19. After months of research, one of the most hotly debated subjects is the significance of vitamin D in the prevention or treatment of COVID-19 [8]. Two ecological studies published recently found inverse relationships between national estimates of vitamin D status and the COVID-19 incidence and death in European nations [9], [10].

Considering the high prevalence of vitamin D deficiency in Iran [11] as well as the high mortality rate associated with COVID-19 in Iran, the current study investigated the blood content of vitamin D and its connection with clinical symptoms in COVID-19 patients admitted to Pastor hospital in Bam, Iran, following disease diagnosis by identifying viral nucleic acid using RT-PCR.

## Materials and methods

### Study population

This study comprised consecutive individuals with COVID-19 who were admitted to Pastor hospital (Bam, Iran) between March 17^th^ and May 31^st^, 2022, with complete vitamin D measurements at admission [12]. The subjects were initially examined in triage and required either ambulatory or in-hospital treatment. According to National Institute of Health guidelines, all patients had moderate to severe illness (moderate illness: evidence of lower respiratory illness during clinical examination or imaging, with an oxygen saturation (SpO_2_) of 94% on air; severe illness: SpO_2_ of <94% on air, a ratio of arterial partial pressure of oxygen to fraction of inspired oxygen (PaO_2_:FiO_2_) of <300 mm Hg, respiratory frequency >30 breaths per minute, or lung infiltrates >50%). A medical history, anthropometric measures, and laboratory testing, including 25 hydroxyvitamin D, were also acquired. To record the outcomes of each patient's hospitalization, the electronic files of each patient were checked. The illness was approached in accordance with Iran National Health guidelines, which were derived from WHO principles. Patients under the age of 18 and pregnant women were excluded. (Tab. 1)

### Ethical considerations

The study protocol was approved by the Ethics Committee of Bam University of Medical Sciences (code of ethics: IR.MUBAM.REC.1400.003) in accordance with the Helsinki Declaration’s ethical standards. All patients provided written informed permission, and their information was kept confidential. 

### Laboratory and clinical measurements

Patients with suspected COVID-19 reported fever, rhinorrhea, sore throat, cough, and likely respiratory distress, especially if they had a positive history of close association with a strongly suspected or proven COVID-19 patient or a travel history to a COVID-19-affected nation or city. The disease was detected using both throat- and nose-swab samples, as well as clinical symptoms, a chest exam, laboratory results, and a reverse-transcription polymerase chain reaction (RT-PCR) test. Based on the evidence or description in the medical records, two experienced radiologists assessed any remarkable radiological findings.

Clinical variables and laboratory measures were obtained at the time of initial evaluation. Physical examination included pulse oximeter saturation (SpO_2_), respiratory rate, temperature and chest CT scan. Laboratory measurements included white blood cell (WBC) count, neutrophil count, lymphocyte count, blood urea nitrogen (BUN), D-dimer, C-reactive protein (CRP) and partial thromboplastin time (PTT). After transferring the blood samples to the laboratory, they were incubated at 37°C for 1–2 hours and then centrifuged at 4,000 RPM for 3 minutes. The obtained serum was separated and analyzed for the serum level of vitamin D using an ELISA (enzyme-linked immunosorbent assay) kit (Pars Azmoun business) via. 

### Vitamin D cut-off

In this case, according to most of the studies [13], the following vitamin D cut-off positions were considered: Vitamin D sufficiency 25(OH)D concentration >30 ng/mL, Vitamin D insufficiency 25(OH)D concentration=20–30 ng/mL, and Vitamin D deficiency 25(OH)D level <20 ng/mL.

### Study interventions

A single oral dose of 200,000 IU of vitamin D3 mixed in a 10-mL peanut oil solution was given to all patients. This dosage is within the recommended range for treating people with 25-hydroxyvitamin D insufficiency [14]. The primary outcome was hospital length of stay, which was defined as the total number of days patients were hospitalized from the time they received vitamin D3 to the time they were discharged. The criteria for patient discharge were as follows: no need for supplemental oxygen in the previous 48 hours, no fever in the previous 72 hours, oxygen saturation >93% without supplemental oxygen, and no respiratory distress. The secondary outcomes were mortality, defined as the number of patients who died during hospitalization, the number of patients admitted to the intensive care unit, the number of patients who required mechanical ventilation and the duration of mechanical ventilation, and serum levels of 25-hydroxyvitamin D (20 and 40 ng/ml). 

### Statistical analysis

Data were analyzed using descriptive statistics (e.g., frequency tables, mean and standard deviation) as well as analytical tests (e.g., Chi-squared, Pearson’s correlation coefficient test, independent t-test and Poisson regression), using SPSS version 27. The probability level of <0.5 was considered to be statistically significant (p<0.05).

## Results

**Figure 1:**
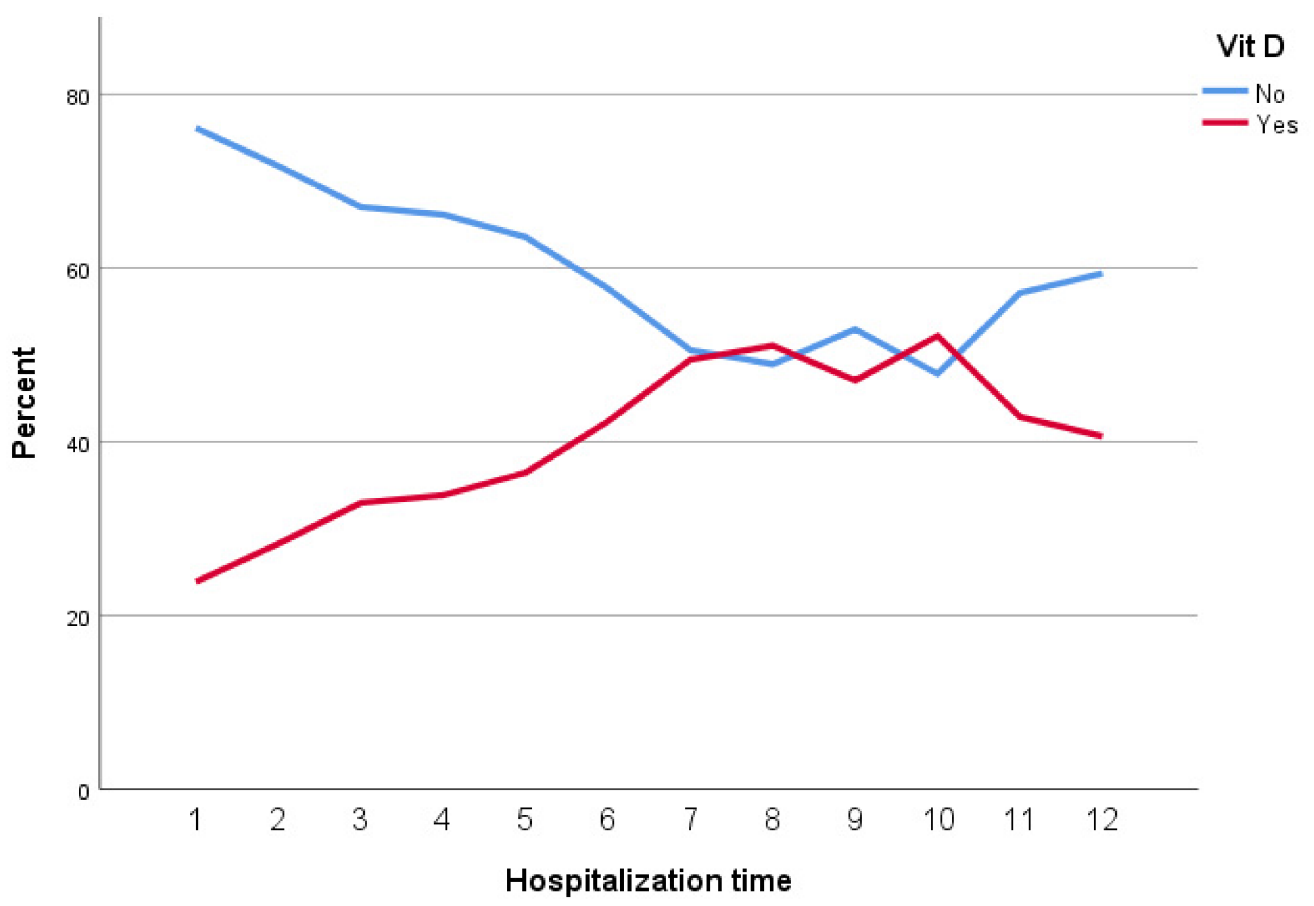
Relationship between vitamin D tablets and hospitalization time

Seventy-one patients with a mean age of 58.2±18.34 years were enrolled, of whom 36 (50.7%) were males. The main clinical presentation was fever (57.7%) followed by cough and respiratory distress. The mean saturation at arrival was 88.3 (STD: 4.78). The summary of the clinical, para-clinical characteristics of the patients, and association with vitamin D levels are presented in Table 1 (Tab. 1), Table 2 (Tab. 2), and Table 3 (Tab. 3). To determine the effectiveness of vitamin D tablets using Poisson regression, the relationship between taking these tablets and hospitalization time was measured. Accordingly, patients who did not take vitamin D tablets were –0.15 times more likely to be hospitalization time than patients who took the Vitamin D pill (Table 4 (Tab. 4)). But as shown in Figure 1 (Fig. 1), after the tenth day, the percentage of people who took vitamin D decreased. Therefore, the results show that vitamin D tablets are effective in accelerating the recovery of COVID-19 disease.

## Discussion

In this study, we provide the experimental data comparing the effects of vitamin D supplementation on the recovery of COVID-19 patients. After being diagnosed with COVID-19 and measuring the level of vitamin D, all people who came to the hospital received a vitamin D supplement. Vitamin D insufficiency/deficiency is widespread in the population, and several articles have shown that vitamin D deficiency is associated with serious diseases COVID-19 [15], [16], [17]. 

Many researchers report that vitamin D deficiency is associated with increased severity of COVID-19 disease, from asymptomatic to symptomatic to respiratory failure, need for mechanical ventilation and death. In the study by Radujkovic et al. [18], the mean vitamin D level in hospitalized patients was 36.5 nmol/L, while in our study, the average vitamin D level is 32.22 nmol/L. People with low vitamin D levels were more likely to require invasive mechanical ventilation and had a higher mortality rate. The study by Pizzini et al. [19] analyzed the relationship between vitamin D and clinical symptoms and progress of COVID-19. In that study, patients infected with SARS-CoV-2 underwent an 8-week follow-up. Finally, Pizzini et al. [19] found that although vitamin D deficiency was common among patients, it was not a determining factor for the severity of the disease and was not related to chest CT changes or abnormalities shown in lung function tests. According to the findings of Pizzini et al. [19], 21.7% of the intubated patients and 35.2% of the patients with disease symptoms on their CT scan had received vitamin D tablets. In our study, vitamin D supplementation improved intubation, PO_2_ and respiratory rate. Also, the consumption of this vitamin improved the symptoms observed in the CT scan of the patients. 

As studies have shown, vitamin D should play a role in SARS-CoV-2 infection as well as in many respiratory infectious diseases. The protective effect of vitamin D in reducing the infection of COVID-19 in patients with severe symptoms of infection and lung damage is probably because the 25 metabolite (OH) D stimulates the production of surfactants in the alveoli. This hypothesis was confirmed by Pereira et al. [20] in a meta-analysis. In their study, a positive correlation was shown between the concentration of vitamin D and the severity of symptoms, which shows that vitamin D deficiency in these patients is associated with a significantly higher risk of hospitalization and mortality. In the study by De Smet et al. [21], 59% of patients with severe COVID-19 pneumonia who required hospitalization had low vitamin D levels (<50 nmol/L) upon admission, which was associated with a higher mortality rate COVID-19.

In the study conducted by Murai et al., which included 240 hospitalized patients with severe COVID-19, a supplemental dose of vitamin D3 was safe and effective in increasing the level of 25-hydroxyvitamin D, but it did not significantly reduce length of hospital stay or any other clinically relevant outcome compared to the control group [22]. These results were also seen in the study by Amrein et al. [23]. While in our study, patients who did not take vitamin D tablets had a 1.17-times greater chance of increased length of hospitalization compared to patients who took the tablets. However, according to the Figure 1 (Fig. 1), after the tenth day, the percentage of people who took vitamin D decreases. Therefore, the results show that vitamin D tablets are effective in accelerating the recovery of COVID-19. Similarly, a study in Spain showed that administration of high-dose vitamin D significantly reduced the need for hospitalization [24], and a double-blind clinical trial reported that among hospitalized patients, those who received vitamin D had. Were hospitalized for shorter periods, spent less time in the ICU and had less need for ventilator support terms of hospitalization, the duration of ICU and the need for ventilator support [25]. 

We investigated the effect of different drugs (Remdesivir, Favipiravir, Dexamethasone, and Actemra) in combination with vitamin D or alone on patients with COVID-19. There was no significant difference in the level of vitamin D between drug groups. However, some studies have reported that dexamethasone increases the effects of 1,25-dihydroxyvitamin D3 by increasing the transcription of the vitamin D receptor, or that vitamin D and remdesivir can combat COVID-19 through a synergistic effect [26]. 

## Limitations

The study was conducted in a single treatment center. Therefore, it is recommended to carry out studies with a larger sample size and investigate the pertinent factors to more accurately evaluate the role of vitamin D in the disease of COVID-19 and its relationship with the severity of the disease. Also, the time elapsed between actual infection and admission was not considered, and quantitative variables were measured only after the patient was admitted to the hospital. This may affect chemical markers of inflammation.

## Conclusion

Our study showed that vitamin D administration significantly reduced the need for intubation, PO_2_, and respiratory rate in patients requiring hospitalization due to COVID-19. Vitamin D appears to reduce length of hospital stay, but larger trials with matched groups are needed to provide a definitive answer.

## Notes

### Author contributions

The authors Mortazavi, Khoshnood and Shirani contributed equally.

### Authors’ ORCID

- Seyed Mojtaba Mortazavi: 0000-0003-2376-3898
- Saeed Khoshnood: 0000-0002-5143-3178
- Reza Faraji: 0000-0002-5973-7301
- Rezvan Bagheri Baravati: 0009-0007-0937-9087
- Hakime Khalili: 0000-0002-8962-2417
- Ali Radfar: 0000-0002-6664-2423
- Elham Jalali: 0009-0006-9107-7856
- Maria Nezam Nia: 0009-0006-3363-7990
- Sousan Akrami: 0000-0001-6643-140X
- Maryam Shirani: 0000-0002-9397-1767

### Ethical approval 

This study was conducted after approval by the ethics committee of Bam University of Medical Sciences (code of ethics: IR.MUBAM.REC.1400.003). 

### Funding 

None. 

### Acknowledgments

We would like to thank the student research committee, School of Medicine, Bam University of Medical Sciences, Bam, Iran for their cooperation. Our appreciation goes to the Vice Chancellor for Research Affairs, Bam University of Medical Sciences, Bam, Iran for their executive and financial support.

### Competing interests

All of the authors declare that there are no commercial, personal, political, or any other potential conflicting interests related to the submitted manuscript. 

## Figures and Tables

**Table 1:**
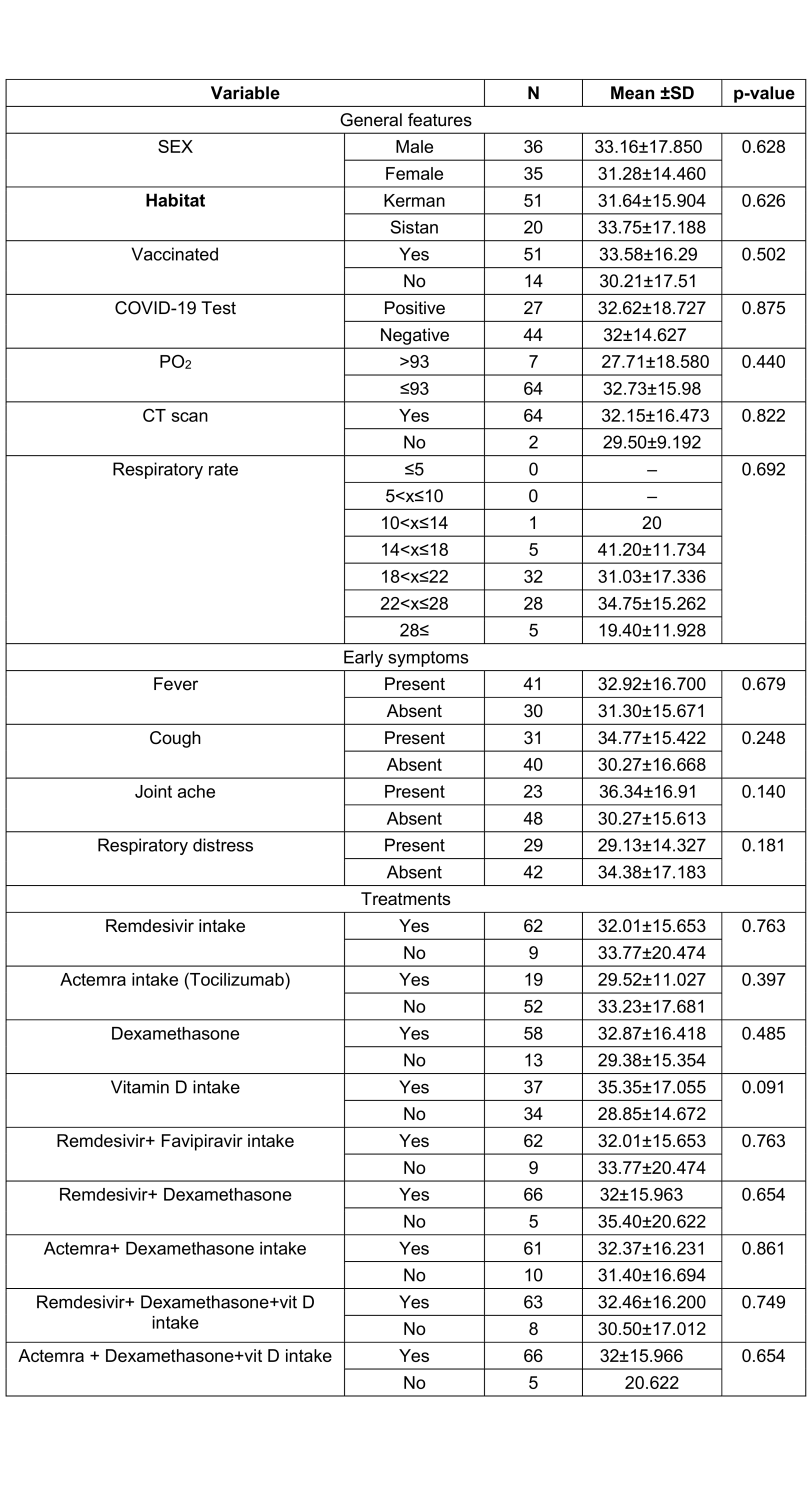
Comparison of clinical features of patients with COVID-19

**Table 2:**
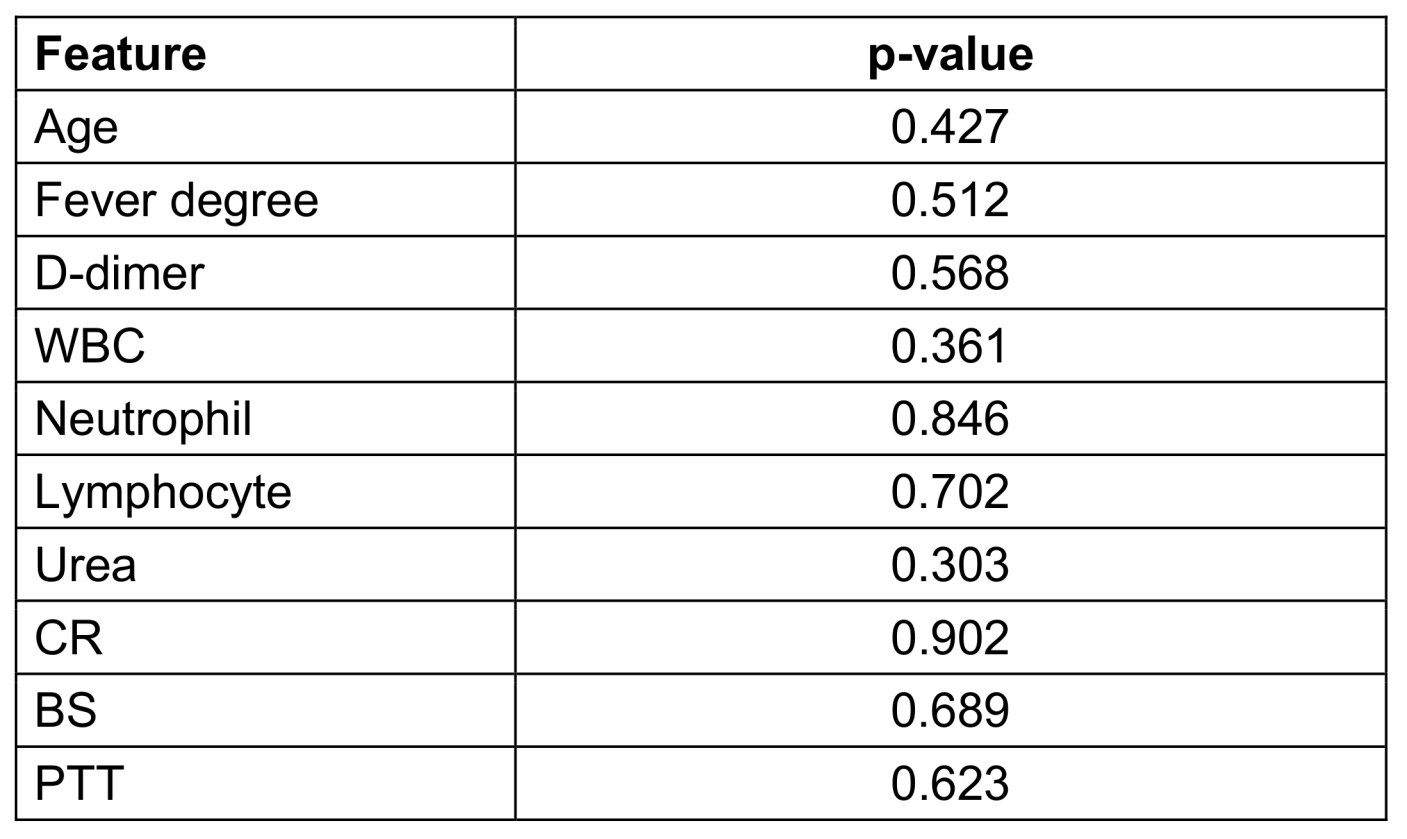
Relationship between vitamin D and laboratory findings in patients with COVID-19.

**Table 3:**
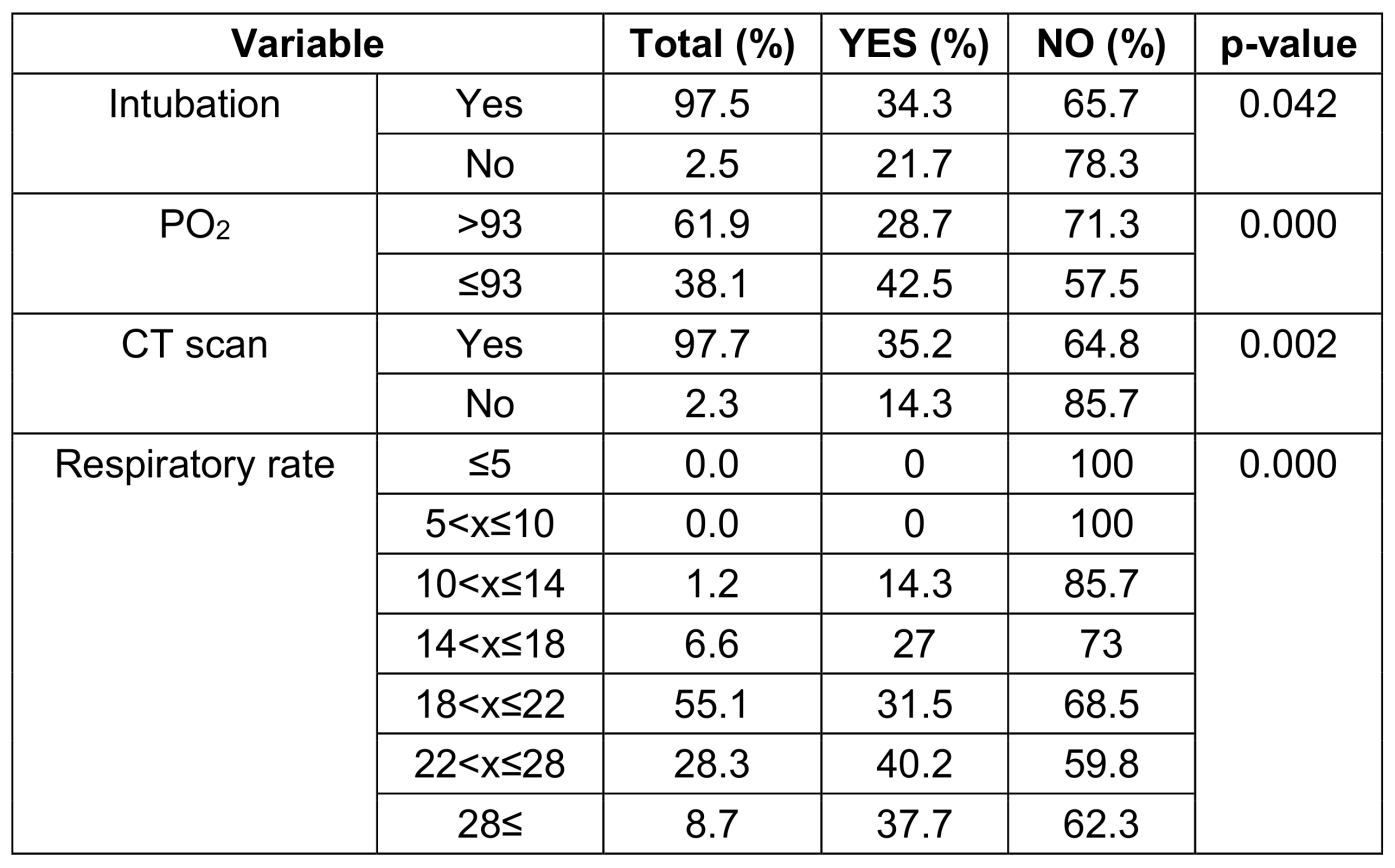
Comparison of the results of taking vitamin D tablets in patients with COVID-19

**Table 4:**
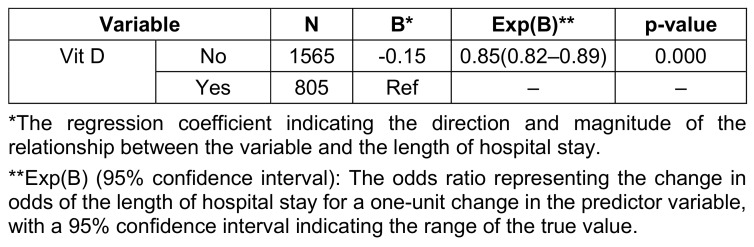
Relationship between vitamin D tablets and length of hospital stay in patients with COVID-19
